# Supplementary material for: Design of optimal labeling patterns for optical genome mapping via information theory
Source: Bioinformatics. 2023 Sep 27;39(10):btad601. doi: 10.1093/bioinformatics/btad601 (PMC10563147; doi:10.1093/bioinformatics/btad601)
Supplement: btad601_Supplementary_Data [file btad601_supplementary_data.zip › si.pdf]

Supplementary Information for: Design of optimal labeling patterns for optical genome mapping via information theory

Yevgeni Nogin[1], Daniella Bar-Lev[2], Dganit Hanania[2], Tahir Detinis Zur[4], Yuval Ebenstein[4,5], Eitan Yaakobi[2], Nir Weinberger[3], and Yoav Shechtman[*,6,7,1]

[1]Russel Berrie Nanotechnology Intitute, Technion, Haifa 320003, Israel
[2]Department of Computer Science, Technion, Haifa 320003, Israel
[3]Department of Electrical Engineering, Technion, Haifa 320003, Israel
[4]Department of Chemistry, Raymond and Beverly Sackler Faculty of Exact Sciences, Tel Aviv University, 6997801 Tel Aviv, Israel
[5]Department of Biomedical Engineering, Faculty of Engineering, Tel Aviv University, 6997801 Tel Aviv, Israel
[6]Department of Biomedical Engineering, Technion, Haifa 320003, Israel
[7]Lorry I. Lokey Center for Life Sciences and Engineering, Technion, Haifa 320003, Israel
[*]Corresponding author: Yoav Shechtman (yoavsh@bm.technion.ac.il)

September 25, 2023

S1 Selection of optimal bin size

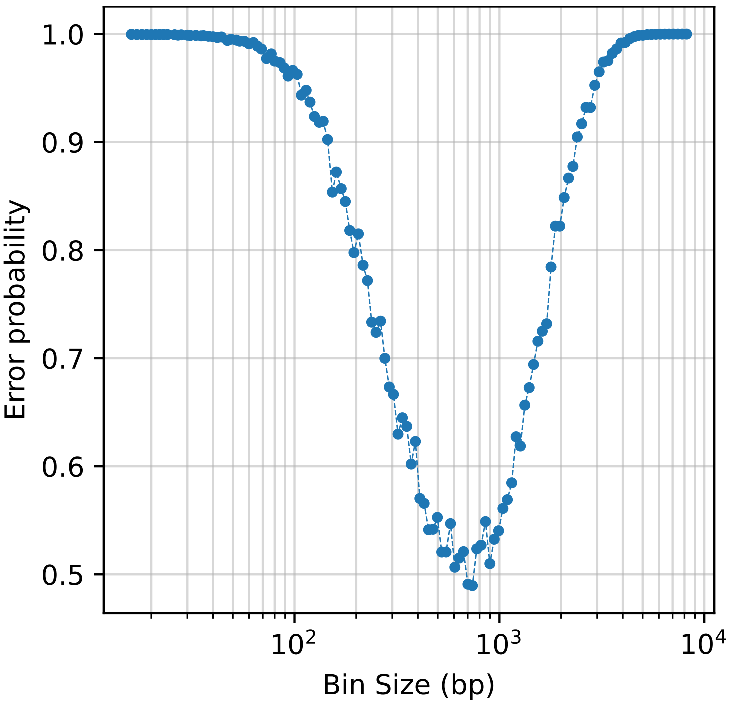

Figure S1: Selection of optimal bin size. To enable the use of the DMC model, the bin size should be larger than the label localization error margin of the localization microscopy method used. If this is not the case, the information in neighboring bins is not independent. To make the assumption of DMC as valid as possible, we optimize the predicted theoretical error probability by varying the bin size. This ensures the tightest possible upper bound on the error probability compared to a decoder (or OGM aligner) that is not limited to binning the labels, the genome sequences, and which does not assume a DMC. The computation of the error probability is done with the same parameters as in Figure 3, for the human genome (DNA fragment length of 50kb, labeled at the pattern CTTAAG). Except that for each bin size, the labeling detection likelihood $p_{y|x}$, as well as p_x, are computed for the specific bin size (Table 1, Section 2.1.2). When the bin size is too low, the localization error of labels relative to the pattern position makes the bins dependent statistically, increasing the label count estimation error and the error probability. When the bin size is too large, the DMC assumption is valid, but too much information is lost, as the number of bins (or codeword length n) is reduced, and the error probability increases. The plot shows that the optimal value is around 1kb, which is the value we used for the bin size B in this work.

S2 Random genome sequence analysis

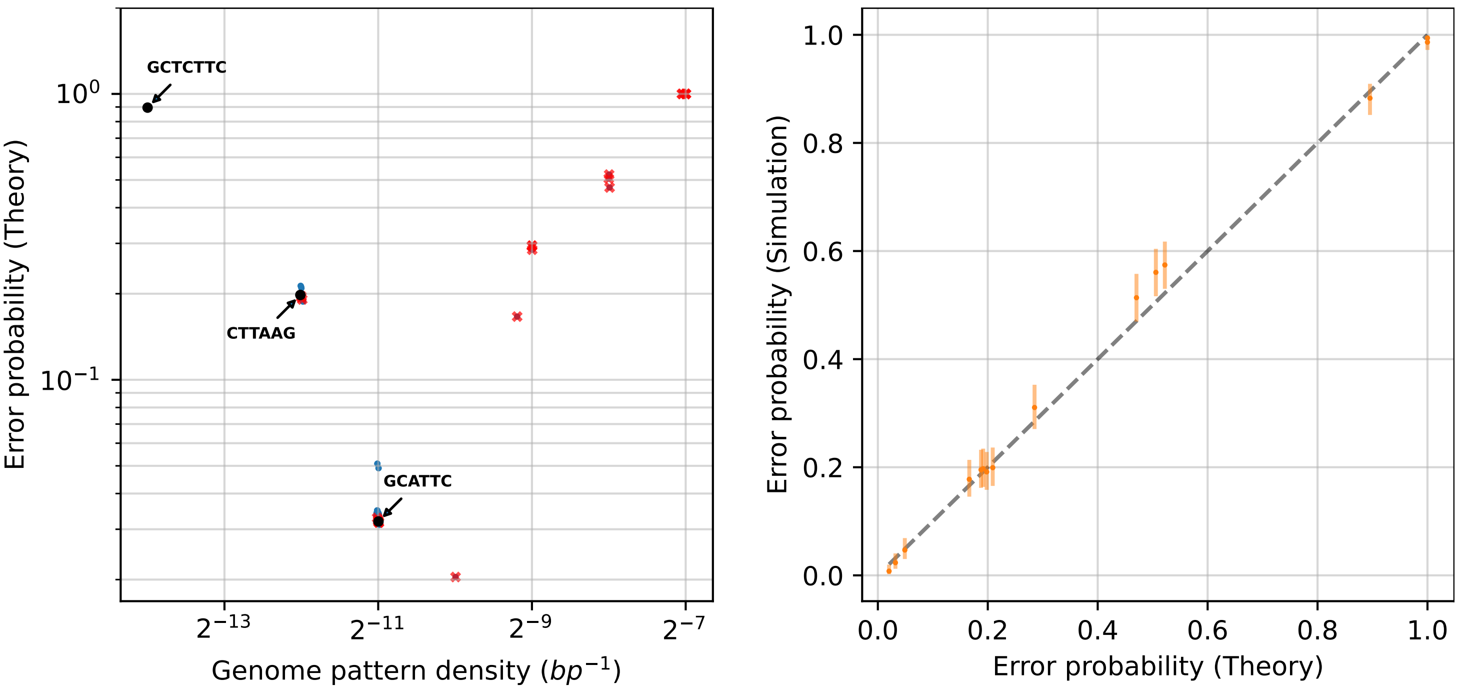

Figure S2: Error probability vs Pattern for a random genome. Here, a random genome sequence of length 10^8 bp was used. The same patterns as in Figure 4 are shown. As expected for a random sequence, all palindromic 6-letter patterns over $\{A, C, G, T\}$ have an approximate density of 4^{-6}, while the non-palindromic patterns have twice the density, as both the ordinary and the reverse complement of the pattern are counted.

S3 Bacterial species

	Organism	Accession	Sequence ID	Sequence Length
0	Citrobacter koseri ATCC BAA-895	GCF_000018045.1	NC_009792.1	4720462
1	Escherichia coli str. K-12 substr. MG1655	GCF_000005845.2	NC_000913.3	4641652
2	Klebsiella pneumoniae subsp. pneumoniae HS11286	GCF_000240185.1	NC_016845.1	5333942
3	Mycobacterium tuberculosis H37Rv	GCF_000195955.2	NC_000962.3	4411532
4	Proteus mirabilis HI4320	GCF_000069965.1	NC_010554.1	4063606
5	Pseudomonas aeruginosa PAO1	GCF_000006765.1	NC_002516.2	6264404
6	Salmonella enterica subsp. enterica serovar Ty...	GCF_000006945.2	NC_003197.2	4857450
7	Staphylococcus aureus subsp. aureus NCTC 8325	GCF_000013425.1	NC_007795.1	2821361

Table S1: Selected bacterial species used in this work. These were selected as they are common in clinical samples and are known to be pathogenic.

S4 Comparison table for selected patterns

	Pattern	Error probability	Genome pattern density (bp^{-1})
0	CCGG	0.056730	0.001501
1	GCAGTG	0.056941	0.000960
2	GGATC	0.078198	0.000983
3	GGTCTC	0.108176	0.000538
4	GACTC	0.112301	0.001273
5	GAGTC	0.112305	0.001273
6	GCAATG	0.136921	0.000465
7	GASTC	0.145349	0.002546
8	GAATGC	0.162584	0.000502
9	GCATTC	0.162584	0.000502
10	GCWGC	0.176059	0.003426
11	GGATG	0.216034	0.001684
12	GCNGC	0.223953	0.004022
13	GTCTC	0.315816	0.002147
14	CTTAAG	0.543502	0.000208
15	CGTCTC	0.576196	0.000209
16	CCWGG	0.596975	0.006383
17	CCNGG	0.647643	0.007523
18	CGCG	0.657824	0.000460
19	GATC	0.784620	0.004662
20	CACGAG	0.822194	0.000127
21	RCCGGY	0.913523	0.000107
22	GCTCTTC	0.984998	0.000061
23	AGCT	0.994101	0.008437
24	CTCGAG	0.998656	0.000039
25	GCCGGC	0.999033	0.000041
26	CGATCG	1.000000	0.000005

Table S2: Special patterns used in the data underlying Figure 4. Error probabilities are given for the human genome, for the following selected patterns: Nicking enzyme recognition sequences (4 letters or longer) from (`http://rebase.neb.com/rebase/azlist.nick.html`), and commonly used patterns in OGM (the enzymes DLE-1, Nt.BspQI, Nb.BsmI with recognition patterns CTTAAG, GCTCTTC, and GCATTC respectively). It is important to note that the label detection likelihood $p_{y|x}$ was estimated for the experimentally available CTTAAG pattern labeling enzyme, and to get more accurate results one should estimate this parameter for each labeling enzyme.

S5 Validity of the binning concept

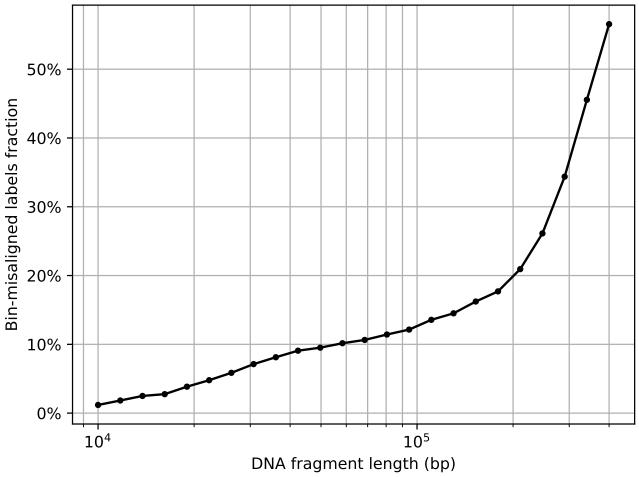

Figure S3: **Bin-misaligned labels fraction**. This figure shows the fraction of bin-misaligned labels per selected DNA fragment length. 32 molecules sampled from the dataset described in Section 2.3 were taken. For each fragment length displayed on the figure, 64 randomly positioned crops of the given length were taken from each molecule in the dataset sample. The fraction of bin-misaligned labels on each cropped fragment were computed as described in Supplementary Section S5. This fraction was averaged over all cropped fragments from all molecules, per examined fragment length, resulting in the line shown.

Here we analyze and discuss the validity of the binning concept in the theoretical model. The presented model assumes a uniform sub-division of both the molecule image and the genome reference to equally sized bins, which are assumed independent. Independence implies that for each label in the reference, its corresponding label in the image is in the same bin index. In practical experimental molecules this is only an approximation. First of all, the optical localization resolution is limited, potentially shifting labels to neighboring bins. Moreover, DNA fragments are not uniformly stretched (as discussed also in Supplementary Section S6). These facts challenge the assumption of bin independence presented in Section 2. In Figure S3 we show that these effects can be neglected, especially for DNA fragments shorter than 100kb, which are the main focus of this work, as they have less than 10% bin-misaligned label fraction.

Now we shall define the metric of bin-misaligned label fraction. Given a DNA fragment image aligned to a genome reference, we divide it and its genome reference to uniformly spaced bins. For each label in the image or the genome reference, one can compute its bin index. Now, the alignment gives a corresponding label on the genome reference for each localized label in the image. Given that information we compute the fraction of labels along the molecule for which the bin-index is different on the image and the genome. This will be the bin-misaligned label fraction, which we show in Figure S3.

S6 Analysis of non-uniform stretching

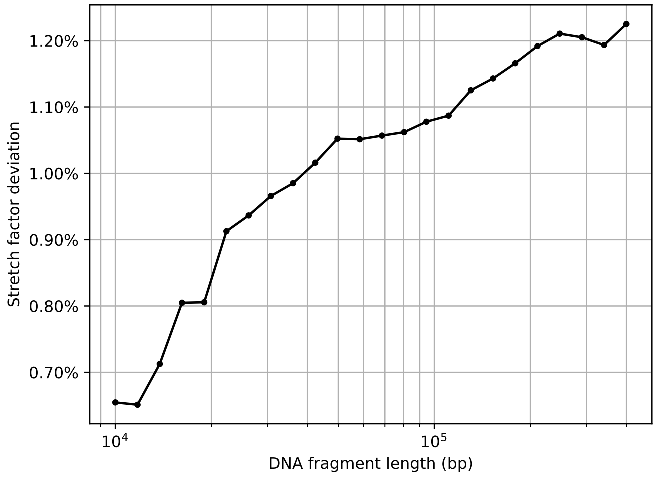

Figure S4: **Stretch factor deviation analysis**. The stretch factor deviation $\tilde{s}$ (equation S1) is shown over a random sample of 32 aligned experimental molecules from the dataset described in Section 2.3. For each aligned molecule, 64 randomly positioned fragments were cropped, for each DNA fragment length examined. The stretch-factor deviation was computed for all cropped fragments. This factor was averaged over all fragments, per examined fragment length, resulting in the line shown.

Here we analyze the non-uniform stretching along molecules in out dataset. Non-uniform stretching that is strong enough, can challenge the binning approach used in the theoretical analysis in this work. In Figure S4 the deviation of the stretch factor along molecules is shown. This figure shows that the deviation is not more than around 1% even for the longest fragments. It is true that for long enough DNA fragments, this stretch factor deviation can accumulate and cause mis-alignment of bins between the molecule image and its corresponding genome reference. Nevertheless, as Figure S3 shows, less than around 10% of the genome reference labels are mis-aligned in terms of their respective bin-indices between the image and genome, for fragments shorter than 100kb, which are the main focus of this work. From these observations, it can be concluded that the effect of non-uniform stretching is negligible for the estimation of mapping error probability as described in the theoretical model, for the fragment lengths addressed in this work.

Now, we shall describe our definition of the stretch-factor deviation. Given an aligned molecule image, we define the local stretch factor at a position q along the molecule, as the derivative of the alignment interpolation function, $s(q) := f'(q)$, where the alignment interpolation function f, is a linear piecewise interpolation for the mapping of aligned label localizations positions to their corresponding aligned genome reference labels. The stretch factor s has units of bp/nm and it represents the genome sequence length covered by a unit of spatial molecule length. If a molecule would be uniformly stretched, the factor s would be constant along the molecule. To be clear, piecewise linear interpolation for a vector of points $(x_0, y_0), (x_1, y_1), \ldots, (x_n, y_n)$ can be represented as a set of linear equations for each interval between consecutive points. The equation for the i-th interval $[x_i, x_{i+1}]$ is given by:

$$f(x) = \begin{cases} y_i + \frac{y_{i+1}-y_i}{x_{i+1}-x_i}(x - x_i) & \text{if } x \in [x_i, x_{i+1}] \\ 0 & \text{otherwise} \end{cases}$$

We define the stretch factor deviation $\tilde{s}$ as the median absolute deviation (MAD) of the stretch factor along the molecule, normalized by the median of the stretch factor along the molecule:

$$\tilde{s} = \frac{\text{MAD}(s)}{\text{median}(s)} \tag{S1}$$

Usage of the median is motivated by the fact that the stretch factor is not necessarily normally distributed, and the MAD is used to avoid the effect of outliers, due to local alignment inaccuracies.

S7 Comparison to the Bionano software

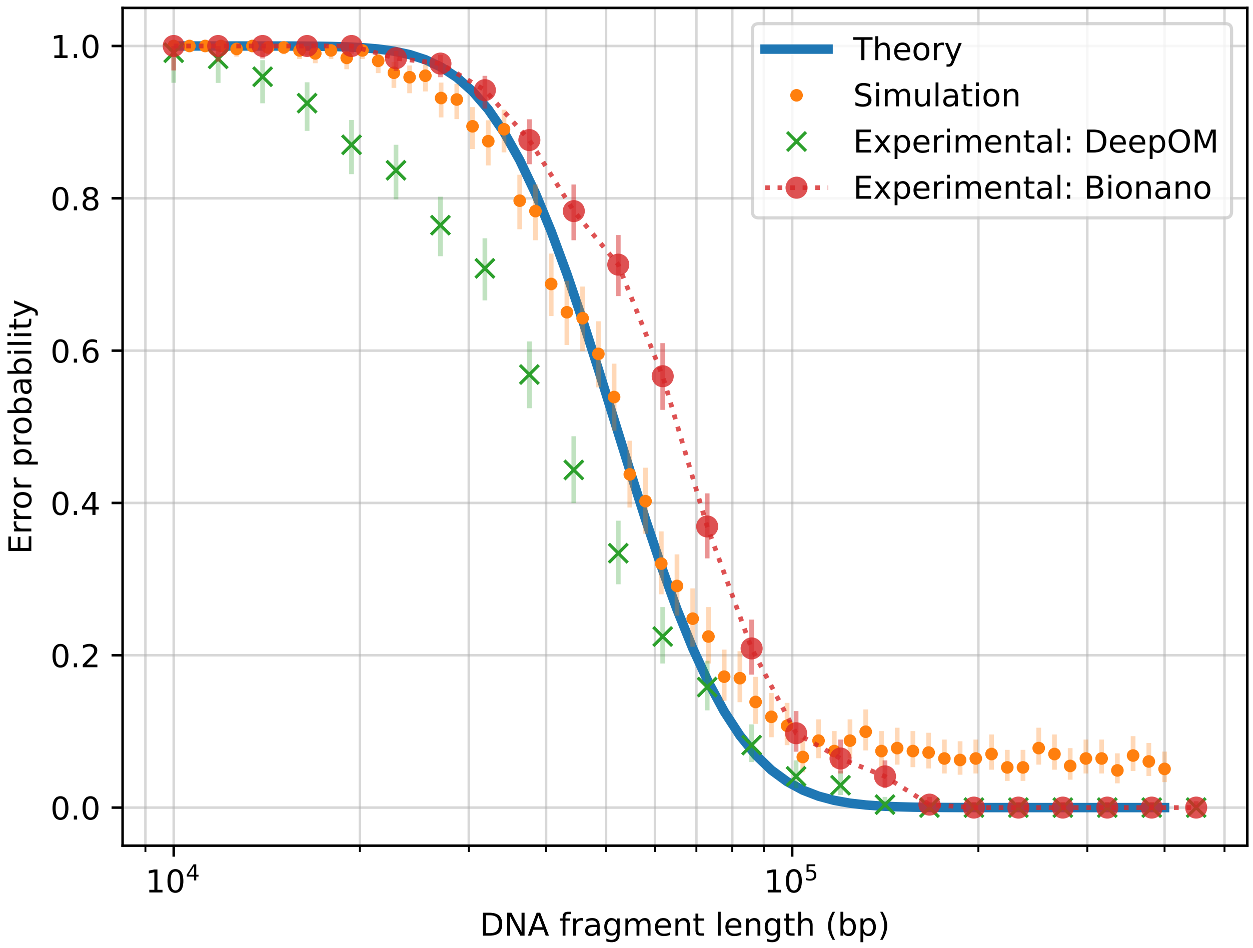

Figure S5: **Experimental validation of the theory vs. Bionano software.** This figure shows the same results as in Figure 3, only overlayed with the results for the Bionano OGM aligner, from Figure 4b in the DeepOM paper (Nogin et al., 2023). As explained in the DeepOM paper, the main reason for the higher mapping errors for the Bionano software, is the fact that the Bionano software is limited in separating too closely spaced labels, reducing the information content available for alignment.

S8 Sensitivity to bin size

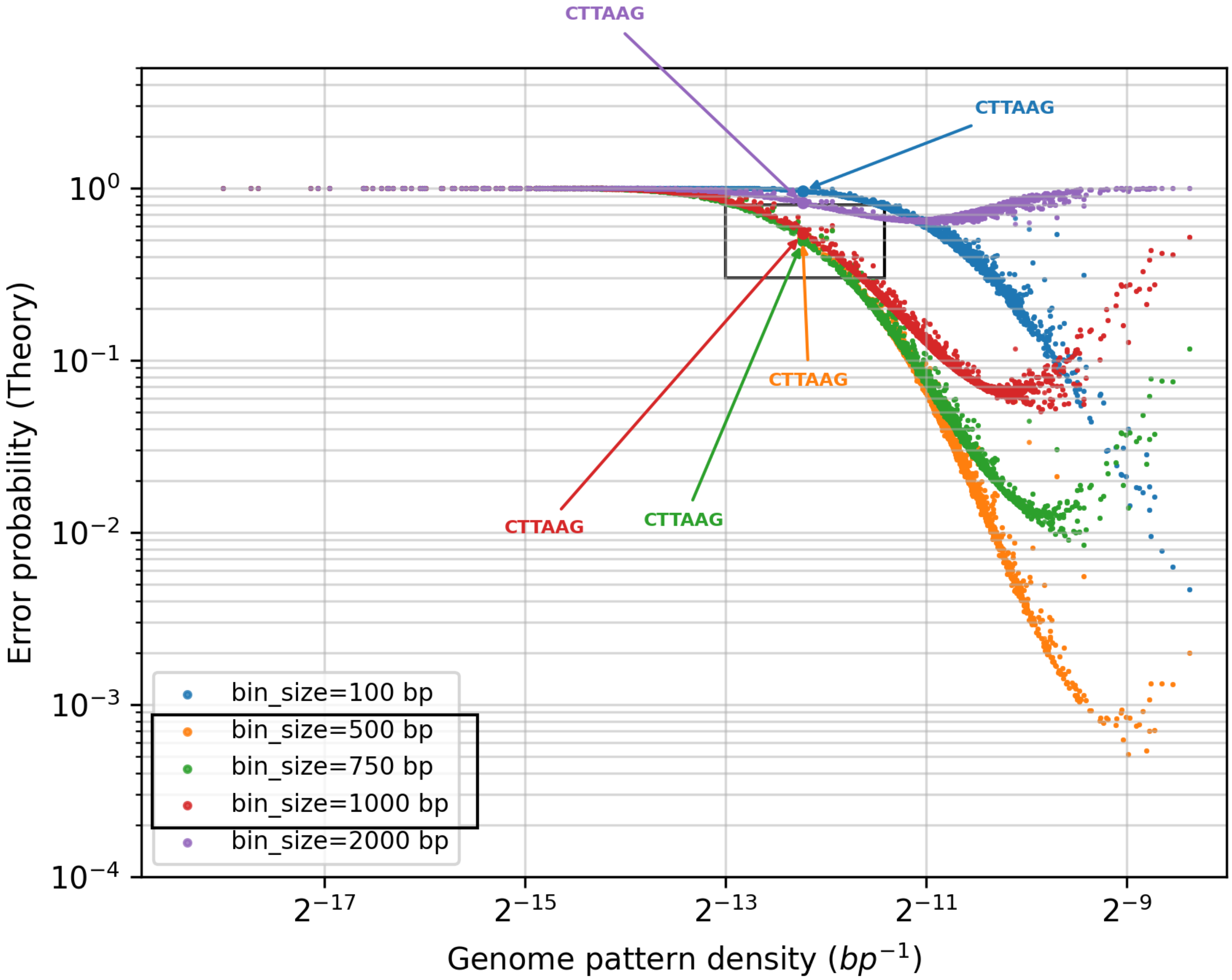

Figure S6: **Sensitivity of the pattern optimization to the bin size.** The bin size is an important parameter of the model and will affect the estimated $p_{y|x}$ and p_x distributions, and thus the estimated error probability. This figure shows the sensitivity of the theoretical model to the bin size parameter. Results similar to Figure 4a are shown (error probabilities for all length-6 labeling patterns for the human genome), only for different bin sizes. Here the figure shows that in the bin size range of 500-1000bp, the error probability is not very sensitive to the bin size, for the pattern available experimentally, CTTAAG. This can be also seen in Figure S1, where the error probability is shown as a function of the bin size. In practice one should optimize for the bin size giving the lowest error probability for each examined labeling pattern as described in Figure S1, and in Section 2.
